# Genomic Evolution of the SARS-CoV-2 Omicron Variant in Córdoba, Argentina (2021–2022): Analysis of Uncommon and Prevalent Spike Mutations

**DOI:** 10.3390/v16121877

**Published:** 2024-12-03

**Authors:** Nadia B. Olivero, Victoria E. Zappia, Pablo Gargantini, Candela Human-Gonzalez, Luciana Raya-Plasencia, Judith Marquez, Lucia Ortiz-Batsche, Mirelys Hernandez-Morfa, Paulo R. Cortes, Danilo Ceschin, Mariana Nuñez-Fernandez, Daniel R. Perez, José Echenique

**Affiliations:** 1 Centro de Investigaciones en Bioquímica Clínica e Inmunología (CIBICI)-Consejo Nacional de Investigaciones Científicas y Técnicas (CONICET), Córdoba X5000HUA, Argentina; victoria.zappia@unc.edu.ar (V.E.Z.); candela.human@mi.unc.edu.ar (C.H.-G.); luciana.raya.plasencia@unc.edu.ar (L.R.-P.); mirelys.hernandez@unc.edu.ar (M.H.-M.); pcortes@unc.edu.ar (P.R.C.); 2 Departamento de Bioquímica Clínica, Facultad de Ciencias Químicas, Universidad Nacional de Córdoba, Córdoba X5000HUA, Argentina; 3 Clínica Universitaria Reina Fabiola, Universidad Católica de Córdoba, Córdoba X5000HUA, Argentina; pablogargantini@curf.ucc.edu.ar (P.G.); judithmarquez@curf.ucc.edu.ar (J.M.); 4 Department of Population Health, College of Veterinary Medicine, University of Georgia, Athens 30692, GA, USA; lortizb@emory.edu (L.O.-B.); dperez1@uga.edu (D.R.P.); 5 Instituto Universitario de Ciencias Biomedicas de Córdoba (IUCBC), Centro de Investigacion en Medicina Traslacional “Severo R. Amuchastegui” (CIMETSA), Consejo Nacional de Investigaciones Científicas y Técnicas (CONICET), Córdoba X5000HUA, Argentina; danilo.ceschin@iucbc.edu.ar; 6 Centro de Química Aplicada, Facultad de Ciencias Químicas, Universidad Nacional de Córdoba, Córdoba X5000HUA, Argentina; mariana.nunez@unc.edu.ar

**Keywords:** SARS-CoV-2, Omicron, HLA alleles, T-cell epitopes, Cordoba, Argentina, epidemiology

## Abstract

Understanding the evolutionary patterns and geographic spread of SARS-CoV-2 variants, particularly Omicron, is essential for effective public health responses. This study focused on the genomic analysis of the Omicron variant in Cordoba, Argentina from 2021 to 2022. Phylogenetic analysis revealed the dominant presence of BA.1 and BA.2 lineages, with BA.5 emerging earlier than BA.4, aligning with observations from other regions. Haplotype network analysis showed significant genetic divergence within Omicron samples, forming distinct clusters. In comparison to global datasets, we identified mutations in the Omicron genomes (A27S, Y145D, and L212I) situated within the NTD region of the Spike protein. These mutations, while not widespread globally, showed higher prevalence in our region. Of particular interest were the Y145D and L212I substitutions, previously unreported in Argentina. In silico analysis revealed that both mutations impact the binding affinity of T-cell epitopes to HLA type I and II alleles. Notably, these alleles are among the most common in the Argentinian population, with some associated with protection against and others with susceptibility to SARS-CoV-2 infection. These findings strongly suggest that these prevalent mutations likely influence the immunogenicity of the Spike protein and contribute to immune evasion mechanisms. This study provides valuable insights into the genomic dynamics of the Omicron variant in Cordoba, Argentina and highlights unique mutations with potential implications for COVID-19 vaccines.

## 1. Introduction

In late 2019, the global landscape was unsettled by the emergence of the severe acute respiratory syndrome coronavirus (SARS-CoV-2), a novel coronavirus that swiftly disseminated worldwide, instigating the coronavirus disease 2019 (COVID-19) pandemic. By September 2024, this had resulted in over 776 million confirmed cases and more than 7 million fatalities worldwide [1]. In Latin America and the Caribbean, the impact of COVID-19 was significant, with more than 25% of global COVID-19 cases reported. Brazil, Argentina, Colombia, and Cuba were among the countries most affected by the pandemic. However, due to differences in poverty levels, healthcare infrastructure, economic volatility, and political decisions, there was noticeable variability in morbidity and mortality rates across different regions [2]. The healthcare systems in Latin America faced serious challenges during the third wave of the COVID-19 pandemic, driven by the Omicron variant, which emerged in December 2021 in Botswana [2]. Despite limited economic resources and capabilities to establish genomic surveillance programs [3], new SARS-CoV-2 variants were identified for the first time in Latin America, some of which became variants of interest or concern [4].

Notably, there was a groundbreaking advancement in the capacity to track genetic mutations that enabled the gradual development of various viral strains [5]. Globally established facilities promptly mobilized to trace the virus’s evolutionary trajectory across distinct regions, patient cohorts, and centers with meticulous examination. This endeavor facilitated an intricate characterization of viral alterations, notably those impacting its interaction with the human host. Moreover, it enabled the surveillance of variants of concern (VOC) delineated by the World Health Organization (WHO) as possessing significant evolutionary advantages, thereby dominating over other co-circulating variants and exerting pronounced public health implications [1].

Argentina has experienced a significant impact from the COVID-19 pandemic, with over 10 million confirmed cases and 130,000 deaths reported so far since its onset [1]. A notable surge in cases was observed between November 2021 and March 2022, largely attributed to the rapid transmission of the Omicron variant [6]. Within the Latin American and Caribbean region, Argentina ranks second in confirmed cases and fourth in mortality rates, highlighting the substantial toll of the pandemic on the country [2].

One prominent aspect of the Omicron variant is its high count of amino acid mutations compared to numerous other SARS-CoV-2 variants. As a result, the Omicron variant exhibits heightened transmissibility compared to its predecessors and possesses the capability to circumvent the immune defenses of the host, thereby facilitating reinfection in both vaccinated and previously infected individuals [7]. Consequently, the rapid dominance of the Omicron variant over other concurrent variants was observed shortly after its emergence, accompanied by a significant increase in its spread rate [8]. Notably, some authors have posited that the disease associated with Omicron differs from those attributed to preceding variants [5].

The Omicron variant harbors at least 32 mutations within the Spike protein [9]. This has raised concerns, as the Spike protein of SARS-CoV-2 is responsible for engaging with the host cell. Comprising S1 and S2 subunits, along with furin protease cleavage sites, this protein contains an N-terminal domain (NTD) and a receptor-binding domain (RBD). The RBD plays a crucial role in engaging with angiotensin-converting enzyme-2 (ACE2); the SARS-CoV-2 receptor presents on the surface of human cells, facilitating virus entry and influencing its transmissibility [9]. Moreover, the Spike protein serves as the primary target for neutralization by convalescent plasma, vaccines, and monoclonal antibodies [10].

In Argentina, the first case of Omicron was reported on 5 December 2021 by the Laboratorio Nacional de Referencia [11]. In Cordoba, it was confirmed on 12 December 2021 [12], which was followed by the third and fourth waves of SARS-CoV-2 infections [6]. A previous report indicated that the third wave was primarily caused by the BA.1 lineage with a low level of clustering, suggesting a relatively low number of introductions but rapid expansion across the country [6]. In contrast, the fourth wave was attributed to the detection of several sublineages of the BA.2 lineage, indicating numerous introductions but limited transmission chains within the country [6].

This study is a retrospective analysis of the genomic evolution of the Omicron variant in Cordoba, Argentina. Samples were obtained from COVID-19 patients at the Reina Fabiola University Clinic (RFUC) from 2021 to 2022. This private medical center provides care to a diverse range of patients across different neighborhoods in Cordoba city, the second largest city in Argentina, with more than 1.4 million inhabitants. Through genomic and phylogenetic analysis, we observed the emergence of the Omicron subvariants mirroring findings in other countries.

Regarding the Spike mutations that help classify SARS-CoV-2 variants, we have identified common substitutions linked to immune evasion. Additionally, we have discovered other noteworthy and prevalent mutations that have not been previously reported in Argentina and have not widely spread worldwide. We have also observed potential changes in the immunogenicity of T-cell epitopes that could affect the immune response to the SARS-CoV-2 virus. We suggest that characterizing the Omicron variants in our region will contribute to understanding the global evolution of SARS-CoV-2 and its potential impact on Spike-based vaccines against this pandemic virus.

## 2. Materials and Methods

### 2.1. Sample Collection

Samples were obtained from different Reina Fabiola University Clinic (RFUC) units, including the outpatient, inpatient, and central emergency units. Either oropharyngeal or nasopharyngeal swabs were independently collected using Dacron swabs with a plastic handle. The swabs were placed in conical tubes containing 1 mL of sterile physiological saline solution and kept at 4 °C for 1–2 h until RNA purification.

Ninety-nine samples were taken in total covering the period between September 2021 and November 2022. Seventy of those samples, obtained from December 2021, revealed the presence of the Omicron variant of SARS-CoV-2 (Table S1). Different neighborhoods of Cordoba city are represented in our sample set.

### 2.2. RNA Purification and Detection by RT-PCR

Ribonucleic acid extraction was carried out using the Highway^®^ DNA/RNA PuriPrep-VIRUS kit (K1501), following the manufacturer’s instructions. RNAse-free water was used as negative control. The RNA was eluted with 50 µL of RNase-free water and stored at −20 °C until further processing. To assess the SARS-CoV-2 RNA levels we used a reverse transcription quantitative polymerase chain reaction (RT-qPCR). For that purpose, the DisCoVery SARS-CoV-2 RT-PCR Detection Kit Rox (DV101; TransGen Biotech Co., Beijing, China) was utilized. This kit targeted the conserved regions of the Orf1ab and N genes as primer and probe binding sites. Moreover, the kit included an endogenous control for detection to monitor the samples, nucleic acid extraction, and PCR processes, thereby reducing the risk of false-negative results.

### 2.3. Nanopore DNA Sequencing

We used the SARS-CoV-2 sequencing protocol developed by the ARTIC Network (https://artic.network/, accessed on 12 August 2022) based on the Nanopore DNA sequencing platform. We followed the instructions according to Artic protocol V.3, available online (https://www.protocols.io/view/ncov-2019-sequencing-protocol-v3-locost-bp2l6n26rgqe/v3, accessed on 12 August 2022). The library construction was performed as follows: cDNA synthesis was performed using the LunaScript RT SuperMix kit (NEB, E3010). Subsequently, overlapping amplicons of approximately 400 bp were generated with Q5 Hot Start DNA Polymerase (NEB M0493). Two separate reactions were carried out using two primer pools, V4.1 (ARTIC nCoV-2019 V4.1 Panel, IDT). The resulting samples were then combined. For end preparation, the NEBNext Ultra II End Repair/dA-Tailing module (NEB E7546L) was utilized.

Multiplexing was achieved using EXP-NBD104 (barcodes 1–12, ONT) and EXP-NBD114 (barcodes 13–24, ONT), along with Blunt TA Ligase Master Mix (M0367, NEB). The manufacturer’s instructions were strictly followed. All barcoded samples were pooled together and purified using AMPure XP Magnetic Beads (Beckman Coulter, Brea, CA, USA, A63880). Quantification of the samples was carried out using the QuantiFluor ONEdsDNA System (ThermoFisher Scientific, Waltham, MA, USA, Q32851). The ligations of the adaptors were carried out using NEBNext Quick Ligation Reaction Buffer (E6056), adaptor MIX (AMII, ONT), and NEB Quick T4 DNA Ligase. The resulting mixture underwent purification using AMPure XP Magnetic Beads. The final library was loaded into a primed R9 flow cell with a total volume of 75 µL, fitted into a MinION instrument (ONT). The DNA sequencing process was controlled using the MinKNOW software program (version 22.10.10) provided by Nanopore Oxford Technologies [13].

Data analysis was conducted using a pipeline constituted by the Epi2me platform (Version 1.2.5, https://epi2me.nanoporetech.com/, accessed on 4 November 2022) developed by Metrichor Ltd. (Oxford, England), which integrates ARTIC and Pangolin sub-sections (Artic + Pangolin-v3.3.1). Artic software was utilized to assess the depth of coverage for each barcoded sample, allowing for exploration of individual amplicons that may not have been adequately amplified using the two primer pools. We analyzed genomes with more than 90% of the DNA sequence complete, and with a coverage higher than 30X. Pangolin was employed to determine the lineage of each sample. The reference genome used for this analysis was the SARS-CoV-2 virus from Wuhan (Accession number: MN908947).

### 2.4. Phylogenetic Analysis

A total of 405 genomes from the Omicron lineage, obtained in Argentina between December 2021 and July 2024, were randomly selected and downloaded from the GISAID platform [14] (https://www.gisaid.org/, accessed on 20 November 2023) for inclusion in this analysis. We conducted a detailed comparison of the genomic sequences from our 70 Omicron strains against 235 Omicron genomes sourced from major Argentinean cities like Buenos Aires, Rosario, and Mendoza. Genomes obtained from Nanopore sequencing and those retrieved from the GISAID EpiCov database were analyzed using the freely available web tool NextClade (https://clades.nextstrain.org/, accessed on 5 December 2023) [15] for alignment and mutation identification. The genomes were classified by Pangolin lineages and hCoV-19/Wuhan/WIV04/2019 strain was used as a reference.

To determine the closest phylogenetic relationships among the Omicron SARS-CoV-2 genomes, we constructed a maximum likelihood phylogenetic tree using Molecular Evolutionary Genetics Analysis Version 11 (MEGA11) [16]. The Neighbor Joining Algorithm was employed as a statistical method. Furthermore, to analyze the evolution of the Omicron variant over the specified period, we generated a time-scaled tree, which was visualized in iTOL v5 [17]. Haplotype network analysis was conducted using PopART, utilizing the Minimum Spanning Network function, version 1.7 [18]. The prevalence of the SARS-CoV-2 mutations was assessed with Lineage/Mutation Tracker [19] (available at https://outbreak.info/situation-reports, accessed on 2 February 2024), utilizing a database containing 15,080,955 genome sequences from GISAID [14].

### 2.5. Protein Variation Prediction and ACE2–Spike Interaction

To predict the impact of amino acid substitutions on the Spike protein’s function, we employed the SIFT (Sorting Intolerant from Tolerant) program [20]. This software predicts whether an amino acid substitution affects protein function based on sequence homology and the physical properties of amino acids. In this study, we used the Uniprot [21], Swissprot, and TrEMBL [22] databases. Also, to assess the impact of these amino acid substitutions on the protein–protein interaction between Spike and the human ACE2 receptor, we utilized the online tool MutaBind2 [23]. As input data, we used the structural PDB file of the ACE2-SARS-CoV-2 Spike wild-type complex (PDB ID: 6M17) [20].

For visualization purposes, the crystal structure of the Spike protein (PDB ID: 6vsb) and the N-terminal domain (PDB ID: 712c) was analyzed using the ChimeraX software (version 1.8) [24]. The three residues that were the focus of this study were highlighted in different colors.

### 2.6. T-Cell Epitope Prediction

The T-cell epitope prediction was carried out using the Wuhan Spike protein sequence (Gene ID: 43740568) and its Y145D and L212I mutant versions. Initially, we selected human leukocyte antigens (HLA-A, HLA-B, HLA-C, DRB1, and DQB1) that are reported to be present in more than 5% frequencies in the Argentinian population [25]. Utilizing the IEDB Analysis resource (Tepitool) [26] with low number of peptides default settings, we predicted the binding sites for 9-mer peptides in the case of class I HLA molecules and 15-mer peptides for class II HLA molecules, and removed duplicated peptides. These preliminary results were confirmed with NetMHCpan 4.1 tool [27] for class I HLA using with the standard settings (strong binder % rank 0.5, weak binder % rank 2, and 9-mer peptides). For class II HLA, results were confirmed with the NetMHCIIpan 4.3 tool [28], also using the standard settings (strong binder % rank 1, weak binder % rank 5, and 15-mer peptides).

### 2.7. Statistical Analysis

To evaluate recombination events, we uploaded our data to the Splitstree software (version 4-19-2) [29] and performed a Phi test. Different bioinformatic software programs were used as indicated in this manuscript and according to developer instructions.

## 3. Results

### 3.1. Identification of the SARS-CoV-2 Variants Circulating in Cordoba City, Argentina

To determine the evolutionary dynamics of the SARS-CoV-2 variant in Cordoba City, Argentina, respiratory samples were obtained from COVID-19 patients attended at the Reina Fabiola University Clinic (RFUC). Nanopore sequencing technology was employed for the genomic analysis of these samples [30]. During September–October 2021, before the emergence of the Omicron variant, 26 genomes were analyzed, and they belonged to three SARS-CoV-2 variants that co-circulated in Cordoba distributed as follows: 6 to Delta, 6 to Lambda (C.37), and 14 to Gamma (P.1, P.1.2, P1.14, and P.1.15).

**Figure 1:**
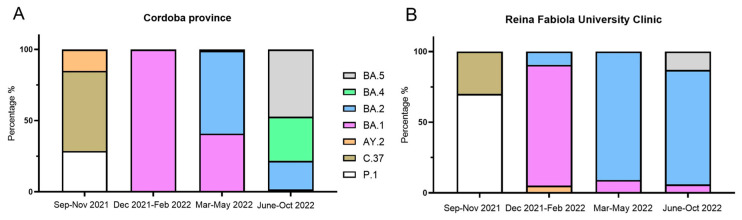
Percentage distribution of the SARS-CoV-2 lineages at different time periods: (**A**) In 731 samples of Cordoba province obtained from GISAID. (**B**) In our 99 samples obtained from the Reina Fabiola University Clinic.

A total of 99 samples were collected from COVID-19 patients from September 2021 to November 2022 (70 of those obtained from December 2021) revealed the presence of the Omicron variant (Table S1). We compared the SARS-CoV-2 lineages found at the RFUC in Cordoba City with those circulating throughout the entire Cordoba province during the same timeframe. This analysis aimed to evaluate how well our samples from the RFUC represent the broader viral landscape in the region. Sequence data from 731 Omicron genomes, which were obtained in the Cordoba province and sourced from GISAID [14], were compared with those collected from the RFUC. We found a prevalent presence of Lambda (P.1) and Gamma (C.37) sublineages during the pre-Omicron period. As described in most countries, these lineages experienced a notable decline in December 2021 that coincided with the appearance and subsequent predominance of the Omicron variant (Figure 1).

During the initial phase of Omicron’s emergence, the BA.1 lineage predominated both regionally and at the RFUC. Subsequently, we detected the first strains of the BA.2 variant at the RFUC, which then showed a clear predominance of BA.2, followed by a displacement caused by the BA.5 variant. At that time, we observed a notable lack of the BA.4 subvariant compared with the subvariants circulating in the province of Cordoba (Figure 1). The potential reasons for this difference will be discussed later.

### 3.2. Phylogenetic Analysis of the Omicron Strains Circulating in Different Regions of Argentina

**Figure 2:**
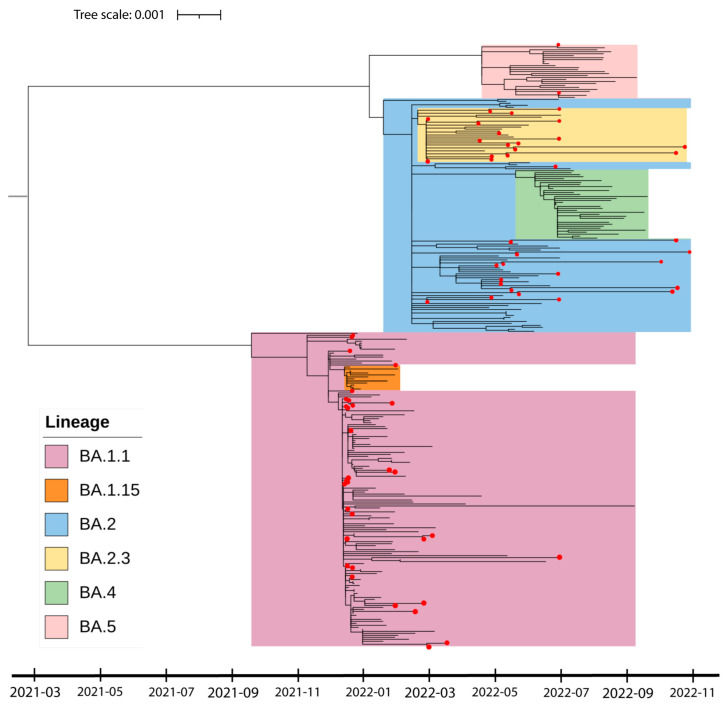
Phylogenetic analysis of the Omicron variant genomes sampled in Argentina. In this study, we compared our Omicron genomic sequences with 235 Omicron genomes (from the GISAID EPICoV database) sampled in Argentina and collected between December 2021 to November 2022. Our samples isolated at the RFUC are indicated with red dots, while the others correspond to the GISAID sequences sampled from the rest of the country. Branch lengths represent genetic distance in terms of substitutions per site, with bootstrap values (500 replicates) indicating branch support. Colors represent different clades of the phylogenetic tree.

After establishing the patterns of SARS-CoV-2 variant spread in Cordoba city and comparing them to the provincial trends, we narrowed our focus to the Omicron variant’s dissemination across Argentina. To achieve this, we conducted a phylogenetic analysis of the 70 Omicron genomes obtained at the RFUC and compared them with 235 genomes obtained from strains circulating in various regions of the country. The integration of these external genetic sequences was instrumental in enhancing our understanding of the Omicron variant’s genetic landscape in Argentina. This approach enabled a shift from a localized to a national perspective. By aligning GISAID genomes with our lab’s sequencing timeframe, we ensured data comparability and minimized temporal biases in viral evolution. The combination of methodological rigor and random nationwide genome sampling strengthened our analysis. The resulting maximum likelihood tree visualized the complex evolutionary pathways and genetic divergence of the Omicron variant in Argentina, offering a deeper understanding of its dynamics (Figure 2). Time-sensitive phylogenetic analysis supported the categorization of SARS-CoV-2 Omicron sequences into two primary clades, BA.1 and BA.2, each displaying heightened genetic divergence.

In November 2021, the BA.1 clade was predominant in Argentina. However, the BA1.15 subclade, which had spread to other cities, was not detected among the RFUC samples. In February 2022, there was a noticeable introduction of the BA.2 subvariant and other related strains, gradually replacing the earlier BA.1 variants. Our phylogenetic analysis revealed the presence of BA.5 genomes in April 2021 before the emergence of BA.4 in May 2021. In the RFUC samples, we identified a few genomes belonging to the BA.5 subvariant, but we did not find any samples corresponding to BA.4 (Figure 2). These results indicate that the dynamic evolution of the Omicron sublineages observed in our sample set differs from that reported in the rest of Argentina. To ensure an up-to-date representation of viral evolution, 170 Omicron genomes from Argentina, covering the period from late 2022 to mid-2024, were added from the GISAID EPICoV database to the phylogenetic tree (Figure S1). Here, we observed the emergence of the XBB variant in February 2023, as described in Latin America and worldwide [2].

**Figure 3:**
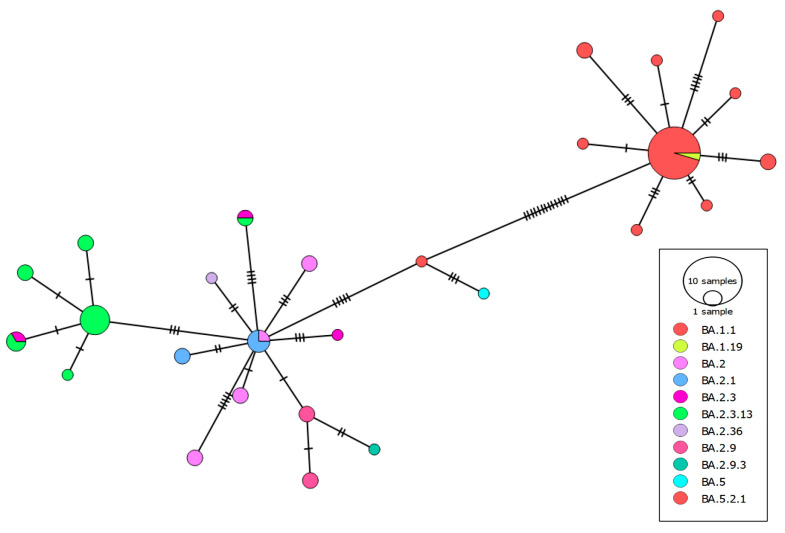
Haplotype network showing the genetic relationship or distances between the 70 Omicron samples analyzed from COVID-19 patients in Cordoba. Nodes in the network represent observed haplotypes, connected by edges indicating genetic similarity. The colors represent different lineages. Hash marks indicate the number of mutations, and the size of the circle represents the number of observed haplotypes.

To graphically represent the genetic diversity within the Omicron population analyzed, an assessment of haplotype networks was conducted using the selected genomic sequences, leveraging genome-wide single-nucleotide variations (SNVs). The results revealed a significant genetic divergence within the cohort of 70 Omicron genomes, illustrating distinct clusters formed by samples sharing the same lineage (Figure 3). The analysis of the haplotype network revealed discernible patterns of genetic similarity and divergence, identified as BA.1, BA.2, and the intermediate BA.5, representing distinct sublineages of the Omicron variant.

Notably, BA.5 occupied a central position between BA.1 and BA.2, suggesting a genetic relationship indicative of the evolutionary interplay between the two major lineages and the potential for genetic exchange between divergent viral populations, as described in other countries [31]. Although the probability of recombination events in a group of samples coming from the same hospital is low, it cannot be completely dismissed. Using Splitstree [32], we performed a Phi test and did not find significant evidence of these events (*p*-value = 0.095) confirming that the evolution from the BA.1 to BA.2 subvariant occurred without recombination events between the SARS-CoV-2 genomes.

### 3.3. Characteristics of Omicron Mutations

This study aimed to examine the Omicron mutations present in SARS-CoV-2 strains, particularly focusing on Spike substitutions. Through genomic analysis, a total of 59 mutations were identified, with the majority occurring in Spike (52%) and Orf1a (22%), and lesser impacts were observed in Orf1b (6.7%), N (6.7%), Orf3a (5%), M (5%), and Orf6 (1.6%). The Sorting Intolerant from Tolerant (SIFT) algorithm was utilized to predict the potential effects of amino acid substitutions on protein function [20]. Of the total mutations, 34 were predicted to be deleterious, while the rest resulted in neutral substitutions (Table S1). Selecting the 15 mutations located in the receptor-binding domain (RBD) of Spike (Table S1), we performed in silico analyses using MutaBind2 online tool to assess the impact of these amino acid substitutions on the protein–protein interaction between Spike and the human ACE2 receptor [23]. Most of them (11/15) correspond to either neutral or deleterious mutations that have demonstrated a decreased interaction with the ACE2 receptor (Table S1).

### 3.4. Worldwide Prevalence of the Omicron Mutations Identified in SARS-CoV-2 Strains Circulating in Cordoba

**Figure 4:**
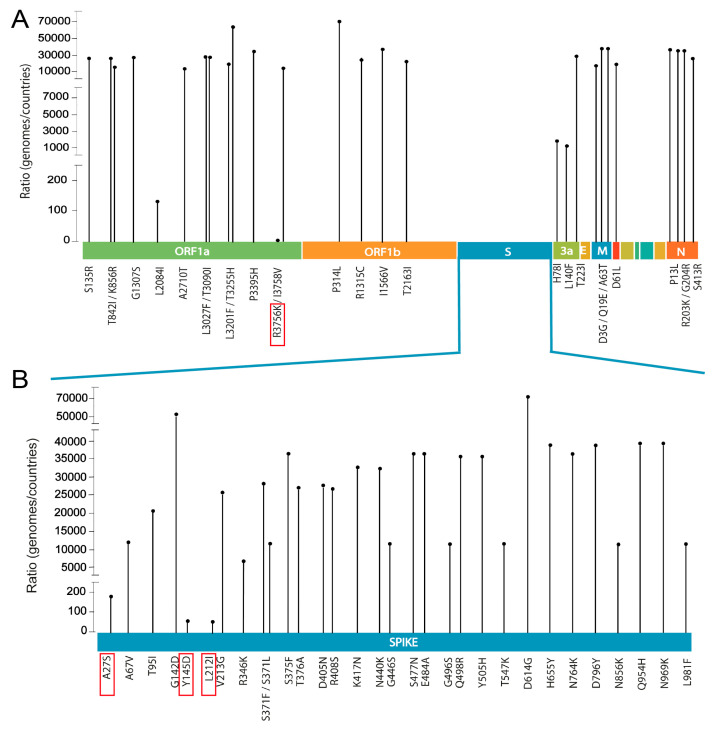
Global prevalence of Omicron mutations in all SARS-CoV-2 proteins. (**A**) Amino acid substitutions across all SARS-CoV-2 proteins; (**B**) Spike mutations. The *y*-axis represents the ratio of SARS-CoV-2 genomes containing each mutation relative to the number of countries reporting the mutation. Vertical lines indicate amino acid mutations in different genomic regions, with mutations highlighted in red representing those with low global prevalence.

To assess the worldwide prevalence of individual mutations found in the Omicron genomes associated with SARS-CoV-2 strains present in Cordoba, we employed the Lineage/Mutation Tracker tool developed by GISAID [14]. To gauge the extent of dissemination, we calculated a ratio by dividing the number of SARS-CoV-2 genomes containing each mutation by the total number of countries where these mutations were reported (#genomes/# countries) (Figure 4), as described [33].

The majority of mutations identified in this study had a global distribution. However, the mutations R3756K (Orf1a), A27S, Y145D, and L212I (Spike) presented limited dissemination on a global scale, with a cumulative prevalence of less than 0.5% (Table S2). Conversely, within the samples analyzed, the R3756K (Orf1a) mutation was detected in 3% (6% in BA.2 samples), the L212I (Spike) mutation in 7% (16% in BA1.1 samples), the A27S (Spike) mutation in 35% (74% in BA.2 samples), and the Y145D (Spike) mutation in 35% (80% in BA1.1 samples) of cases (Table S2). These results suggest a notably high prevalence of these mutations within the studied population, indicating potential advantages for their transmission in the Cordoba region.

### 3.5. The Y145D and L212I Spike Mutations Induce Changes in T-Cell Epitopes

Interestingly, the three Spike mutations (A27S, Y145D, and L212I) identified in our samples, despite their low global occurrence, are notably prevalent in our sample set. These mutations are situated within the N-terminal domain (NTD) of the Spike protein. Analysis using the SIFT algorithm revealed that the A27S and Y145D mutations are likely to be deleterious, suggesting a possible adverse effect on protein features (Table S1). Overall, in the Omicron variant, it has been described that several of these mutations impact the antigenic properties of the Spike protein and contribute to the evasion of the immune response by the SARS-CoV-2 virus [7].

**Figure 5:**
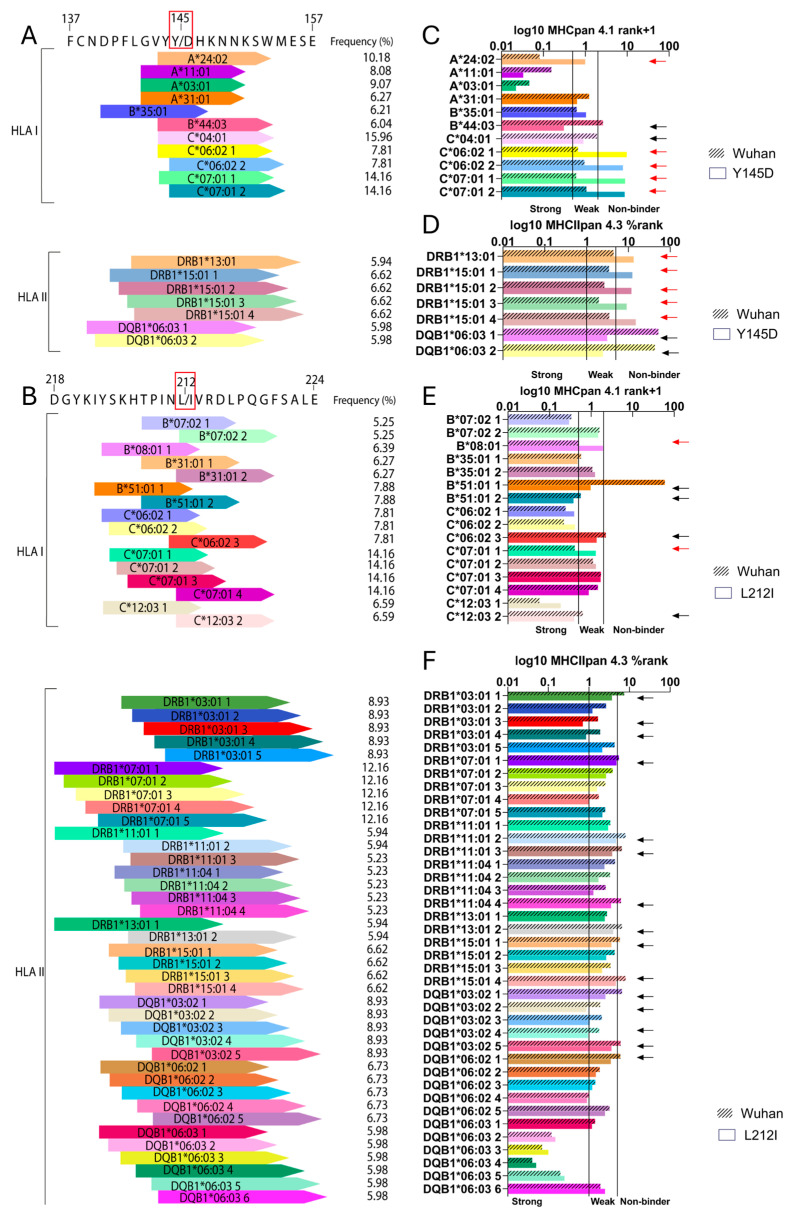
T-cell epitope changes induced by the Y145D and L212I mutations in the Spike protein. (**A**) T-cell epitope distribution between positions 137 and 157 of the Spike protein. (**B**) T-cell epitope distribution between the positions 218 and 224 of the Spike protein. (**C**) Predicted binding affinity of HLA-I molecules between positions 137 and 157 of the Spike protein. (**D**) Predicted binding affinity of HLA-II molecules between positions 137 and 157 of the Spike protein. (**E**) Predicted binding affinity of HLA-I molecules between positions 218 and 224 of the Spike protein. (**F**) Predicted binding affinity of HLA-II molecules between positions 218 and 224 of the Spike protein. Black arrows indicate alleles where the mutation increases the predicted binding affinity, while red arrows indicate cases where the mutation reduces the predicted binding affinity.

One method to analyze the potential impact of SARS-CoV-2 on immunogenicity is to determine the affinity of T-cell epitopes recognized by HLA alleles in a specific human population [34,35]. Individual mutations that affect this interaction could partially alter the affinity for specific T-cell epitopes, consequently altering immune protection against SARS-CoV-2 [36]. Here, we assessed the impact of these three mutations on the T-cell epitope binding capacity. We examined potential T-cell epitopes expected to be recognized by specific Human Leucocyte Antigen (HLA) class I molecules (HLA-A, B, and C, implicated in CD8+ T-cell epitope recognition) and class II molecules (HLA-DPA1, DPB1, DQA1, DQB1, and DRB1, involved in CD4+ T-cell epitope recognition). The analysis was performed considering the HLA alleles that are prevalent in the Argentinian population [25], with frequencies higher than 5%. The HLA alleles utilized in this study are detailed in Table S3. By utilizing the IEDB Analysis resource (Tepitool) [26], together with the NetMHCpan-4.1 for class I HLA and NetMHCIIpan-4.3 for class II HLA molecules, we identified that the A27, Y145, and L212 amino acids of the Spike protein in the wild-type Wuhan strain are part of various epitope peptides recognized by the HLA alleles included in the analysis (Figure 5 and Figure S1). Notably, the mutations Y145D and L212I exhibited changes in the recognition of T-cell epitope patterns (Figure 5), whereas the A27S mutation showed the same pattern as the wild-type Spike version (Figure S1). It is important to note that the Y145D mutation decreased the affinity, as indicated by an increase in NetMHCpan % ranks of HLA molecules with high prevalence (5–15%) in the studied population, such as HLA-A*24:02, HLA-C*06:02, HLA-C*07:01, HLA-DRB1*13:01, and HLA-DRB1*15:01 (Figure 5C,D). In contrast, the Y145D mutation gained affinity for HLA-C*04:01, HLA-B*44:03, and DQB1*06:03, as indicated by an increase in NetMHCpan % ranks (Figure 5C,D).

The 212 residue of Spike belongs to a hot spot for HLA molecules binding, especially class II HLA. In the case of the L212I mutation, the substitution impairs the interaction of the NTD with HLA-B*08:01 and modifies one of the C*07:01 epitopes (Figure 5E). Contrarily, the mutation increases the binding affinity of HLA-B*51:01, one epitope of HLA-C*07:01, and one of HLA-C*12:03 (Figure 5E). Regarding class II HLA, the L212I mutation in the Spike protein increases the binding affinity of HLA-DRB1*03:01, HLA-DRB1*11:01, HLA-DRB1*15:01, and HLA-DQB1*03:02. Also, the HLA-DRB1*11:01, HLA-DRB1*11:04, and HLA-DQB1*06:02 allele recognition sites in the NTD were altered. Taken together, these findings suggest that the changes in T-cell epitopes induced by the Y145D and L212I mutations in the Spike protein may affect T-cell recognition and the immune response.

We also analyzed the HLA epitope pattern in the mutation R3756K of the Orf1a protein, which was the only non-Spike mutation with high prevalence in our sample set and limited global spread. In this case, no class II HLA epitopes were predicted for either the wild-type or mutated version of Orf1a, while the mutation increased the binding affinity of HLA-A*03:02, HLA-A*11:01, HLA-C*04:01, and HLA-C*06:02. In contrast, HLA-A*21:01 affinity binding was impaired by this mutation (Figure S3).

## 4. Discussion

The rapid spread of the Omicron variant has presented considerable obstacles to public health, leading to a rapid increase in COVID-19 infections on a global scale [37]. Through our retrospective analysis covering the period from November 2021 to October 2022, we observed a significant influence of the Omicron variant on the local patterns of viral transmission.

Upon examining the transmission patterns of Omicron strains in Cordoba City, notable distinctions were identified in comparison to regional data. Specifically, the BA.2 variant was identified in samples from the RFUC in February 2022, preceding its detection in the provincial dataset by a month (Figure 1). This discrepancy is likely attributed to the initial introduction of the variant in Cordoba City, followed by its dissemination to neighboring areas. Notably, Cordoba airport, the second most significant airport in Argentina, resumed operations for domestic and international flights in October 2021 after the pandemic-related restrictions imposed by the national government. This development may have played a pivotal role in facilitating the spread of the Omicron variant within the central region of Argentina.

The examination of evolutionary relationships through phylogenetic analysis identified distinct clades within the Omicron sequences sourced from the RFUC, demonstrating genetic variation that has accumulated over successive periods (Figure 2). The positioning of the BA.5 variant between the BA1 and BA.2 variants suggested a potential evolutionary interaction between these primary lineages, indicating genetic interchange among distinct viral populations. By employing the Phi test, we were able to rule out the likelihood of recombination events during the development of the BA.1 and BA.2 subvariants. The analysis of haplotype networks provided additional insights into notable genetic differentiation, revealing distinct clusters within the Omicron samples (Figure 3). The distribution of lineages observed in the RFUC reflected overarching patterns in Córdoba City, with particular lineages becoming more prevalent over time. Despite the valuable insights provided by this study, limitations, such as the small sample size and retrospective nature, should be considered. The focus on a single medical center may not fully represent the broader population variability in Córdoba. Nevertheless, the findings contribute to our understanding of the Omicron variant’s genomic dynamics

In Argentina, the first wave of COVID-19 occurred between August and November 2020, followed by a second wave from March to July 2021 [38]. Prior research has indicated that the emergence of the BA.1 variant in our country in December 2021 initiated the third wave of COVID-19, leading to a surge in cases in mid-January 2022 and ultimately supplanting the Delta variant by the end of January 2022 [6]. This nationwide study analyzed samples from 14 provinces. However, Cordoba, the second most populated province of Argentina, was not included [6]. Our data concerning the emergence of BA.1, the displacement of Delta, and the fourth wave initiated by BA.2 coincide with those reported by these authors. However, we detected the presence of genomes of BA.5 in RFUC in April 2021 before the emergence of BA.4 in May 2021, with samples from patients across different neighborhoods in Cordoba city (Figure 1 and Figure 2), while the emergence of both lineages was reported as simultaneous in Argentina [6]. This spreading pattern was first observed in South Africa, where the BA.4/BA.5 Omicron variants were first detected. Interestingly, the BA.4 variant emerged one or two months before BA.5 [39,40]. In Latin America, the same pattern was observed in Brazil, Paraguay, Panama, and Mexico [2,41]. In contrast, BA.4 emerged before BA.5 in Chile, Uruguay, Bolivia, and El Salvador [41], while in the US, for instance, both variants were detected simultaneously [2]. Generally, both variants co-circulated throughout most of 2022, although BA.4 was predominantly disseminated in the USA, Austria, Denmark, and the UK, while BA.5 is detected in Portugal, Germany, the USA, and the UK [42].

Therefore, we propose that these differences are caused by various spreading factors, such as geographical distances and passenger traffic, among others, rather than solely by specific mutations that confer certain spreading advantages to the Omicron strains. BA.5 exhibited a higher infection rate compared to its predecessors, including BA.4 and BA.2, particularly among vaccinated or previously infected individuals. This increased transmissibility likely facilitated its rapid spread in various regions [43]. In addition, BA.5 presents mutations like L452R and F486V, which contribute to its immune evasion capabilities [44]. This trait may have allowed for BA.5 to outcompete BA.4 in certain populations.

To date, over fifty mutations have been documented in the evolutionary trajectory of the Omicron variant, with particular attention directed towards Spike mutations owing to their significance in the interaction with the hACE2 receptor. These mutations have bestowed enhanced viral fitness, leading to modifications in the transmission dynamics and pathogenicity of the virus [8]. The analysis of 15 mutations located in the receptor-binding domain (RBD) of the Spike protein revealed that most of these mutations were classified as either neutral or deleterious. Computational assessments indicated that these mutations resulted in diminished binding interactions with the ACE2 receptor (Table S1). Noteworthy among these mutations were the K417N and E484A substitutions, which have been experimentally demonstrated to lower the binding affinity between the RBD segment of the Spike protein and the human ACE2 receptor [8]. Mutations that result in a weak hACE2 binding affinity, a characteristic that should harm viral pathogenesis, may also alter the RBD epitopes that facilitate the antibody evasion capacity. In addition, certain negative effects caused by these mutations could be effectively compensated by epistatic mutations, which are defined as being dependent on previously emerged mutations and that could not be tolerated otherwise [45]. It has been proposed that the driving force behind the evolution of Spike does not solely involve evading antibody neutralization or enhancing ACE2 affinity, but rather relies on maintaining a delicate balance between both mechanisms [46].

In addition to the Spike mutations, we also focused our study on the R3756K mutation localized in Nsp6, a non-structural protein within open reading frame 1a (Orf1a). A recent genomic analysis of Nsp mutations in 91,596 human SARS-CoV-2 whole-genome sequences across 19 variants obtained from GISAID revealed the presence of the ∆3675/3677 in Nsp6 of the Omicron variant, but confirmed the absence of the R3756K mutation [47]. Nsp6 is involved in viral replication, forms heterodimers with Nsp3 or Nsp4, and contributes to the formation of double-membrane vesicles and autophagosomes to compartmentalize this process [48]. It has been reported that mutations in Nsp6 lead to reduced susceptibility to IFN-I treatment in vitro and increase virulence in mice, suggesting that mutations outside the Spike protein affect virus–host interactions and may alter the pathogenesis of SARS-CoV-2 variants in humans [49].

Comparisons with global datasets indicated that most mutations identified in our study exhibited widespread distribution globally. Importantly, the R3756K (Orf1a), A27S, Y145D, and L212I (Spike) mutations displayed limited dissemination globally (Figure 4).

**Figure 6:**
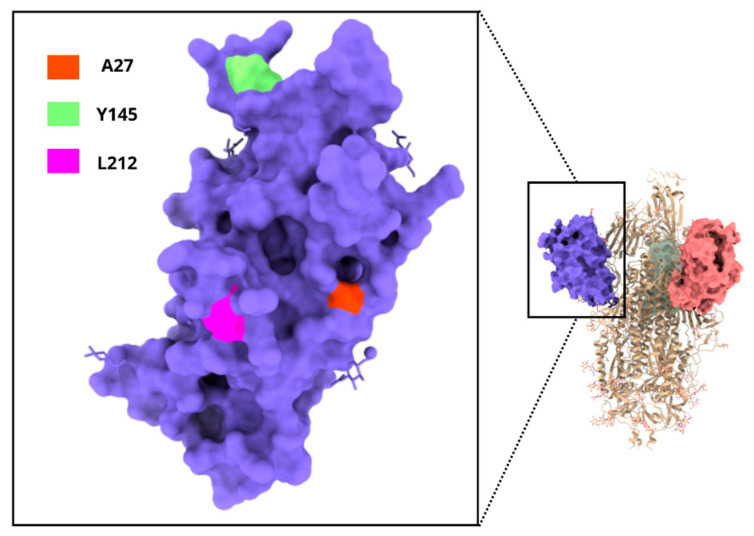
Localization of the A27, Y145, and L212 amino acids in the NTD region of the Spike protein. The structure of the N-terminal domain of Spike domain is colored in blue and the localization of the three residues analyzed is highlighted in different colors.

In this work, these mutations showed an unexpectedly high prevalence among the SARS-CoV-2 genomes from samples obtained in Cordoba, which likely have particular characteristics that have probably affected the dissemination in Cordoba. Three of the highly prevalent mutations, A27S, Y145D, and L212I, are exposed on the surface of Spike (Figure 6), and they are located in the N-terminal domain (NTD), which modulates TMPRSS2-dependent viral entry, fusogenicity, and infectivity of SARS-CoV-2 [50]. More recent Omicron subvariants, such as JN.1 (first detected in Luxemburg/Iceland) and BA.2.87.1 (emerged in South Africa in September 2023), showed more mutations on the NTD than on the RBD [51]. While A27S was present in all the subvariants that circulated after BA.4/BA.5, such as XBB.1.5, XV.1, BA.2.86, JN.1, and BA.2.87.1, the L212I mutation was detected only in BA.2.86/JN.1, and the Y145D mutation was absent in all the subvariants mentioned [52]. This localization could be related to interaction with neutralizing antibodies and immune cell epitopes, and specific mutations on NTD may affect immune evasion that facilitates the Omicron spread [53].

The Spike protein has been extensively investigated as a primary target of immune cells that trigger specific immune responses [54], which can be initiated by the responses to TLR-mediated innate immune cells: T-cells, and B-cells [55,56]. The NTD mutations have mainly been found in three loops falling into the 14–26, 141–156, and 246–260 regions, which constitute the ‘NTD neutralization supersite’, one of the Spike regions where neutralizing antibodies bind [53]. Additionally, Spike exposes other binding sites recognized by T-cells, consisting of short peptides (8–15 amino acids) recognized by HLA-I and HLA-II molecules. Identifying T-cell epitopes is crucial because cytotoxic CD8+ T-cells are essential for clearing SARS-CoV-2 and other intracellular viral pathogens, while CD4+ T-cells are relevant for the coordination with other immune cells against infection and disease [57]. These immune cells also play a primary role in the adaptive immune response and regulate humoral and cellular immunity to SARS-CoV-2 infections, particularly long-term immunity [58]. It has been reported that mutations located within Spike T-cell epitopes showed reduced predicted binding affinity. Spike mutations may induce changes in T-cell epitope recognition, leading to host susceptibility or immune evasion, thereby impacting the clinical severity of COVID-19 infections [34].

In this work, we performed an in silico analysis to determine the putative changes in HLA binding affinities caused by substitutions in Spike. We used a library of HLA alleles that have been commonly identified in the Argentinian population, with frequencies higher than 5%, including class I (HLA-A, B, and C) and class II (HLA-DPA1, DPB1, DQA1, DQB1, and DRB1) molecules [25]. The research identified various HLA alleles associated with epitopes containing the A27, Y145, and L212 amino acids of the Wuhan-1 strain (Figure 5). Analysis of prevalent Spike mutations revealed that while A27S did not affect HLA allele affinity (Figure S2), Y145D and L212I significantly altered T-cell epitope recognition patterns (Figure 5). Remarkably, the alleles HLA-B*51:01, HLA-C*06:02, HLA-C*07:01, HLA-C*12:03, and HLA-DRB1*11:04 were associated with protection against SARS-CoV-2 infection, while the alleles HLA-B*08:01, HLA-B*44:03, DRB1*11:01, DRB1*11:04, DRB1*13:01, DRB1*15:01, DQB1*06:03, and DQB1*06:02 were associated with susceptibility to infection with SARS CoV-2 [59].

It has been reported that an increasing number of Spike epitopes from the Omicron strains present a reduced predicted binding affinity compared to the Delta strains [34]. These changes in the recognition of T-cell epitopes affect host immunogenicity, particularly long-term immunity. In addition, it has been proposed that these variations contribute to determining the clinical severity of acute COVID-19 [34]. The substitutions that negatively impact the immune response against SARS-CoV-2 infections are known as escape mutations and are considered a major, underappreciated factor that directly influences the evolution of this virus in humans [60].

The immune response against SARS-CoV-2 infections is much more complex than the analysis of specific mutations that could affect the recognition of T-cells. In this work, we concluded that the unusual prevalence of Spike mutants in the central region of Argentina, compared to the low distribution worldwide, contribute to a better understanding of the Omicron evolution in our region. These findings underscore the importance of continuous genomic surveillance to track the global spread and evolution of SARS-CoV-2 and to identify unique mutations that may have regional significance. In addition, our findings contribute to the knowledge of the role of NTD in viral infectivity, a region of the Spike protein that exhibits a high number of mutations and appears to be significant as a target for immune cells during the evolution of SARS-CoV-2.

Looking to the future, we consider that genomic surveillance programs should continue in our country to enhance our understanding of the evolution of SARS-CoV-2. Comparative analyses with other geographical regions are essential, as they can provide profound insights into vaccine efficacy and public health. Our work presents a clear example of SARS-CoV-2 mutations that exhibit varying prevalence in relation to the dissemination of specific mutations worldwide. These variations could influence interactions with human immunity and may guide the development of universal vaccines or therapeutic strategies capable of addressing multiple variants.

## Data Availability

All the SARS-CoV-2 genomes generated and presented in this study are publicly accessible through the GISAID platform (https://www.gisaid.org/). Accession numbers are listed in Table S4.

## Supplementary Materials

The following supporting information can be downloaded at: https://www.mdpi.com/article/10.3390/v16121877/s1, Figure S1: T-cell epitope distribution in the A27S mutation. (A) T-cell epitope distribution between the positions 19 and 45 of the spike protein on its wild-type version. (B) T-cell epitope distribution between positions 19 and 45 of the spike protein on the A27S spike mutant; Table S1: Characteristics of the SARS-CoV-2 mutations from the RFUC genomes; Table S2: Times that each mutation was found in different locations; Table S3: HLA alleles used in this study; Table S4: Accession numbers of SARS-CoV-2 genomes publicly accessible through the GISAID platform (https://www.gisaid.org/); Tables S5 and S6: acknowledgments to the contributors of the genomes sequences utilized in this work.
