# Supplementary material for: Genomic Evolution of the SARS-CoV-2 Omicron Variant in Córdoba, Argentina (2021–2022): Analysis of Uncommon and Prevalent Spike Mutations
Source: Viruses. 2024 Dec 3;16(12):1877. doi: 10.3390/v16121877 (PMC11680156; doi:10.3390/v16121877)
Supplement: Supplementary file 1 [file viruses-16-01877-s001.zip › viruses-3237805-supplementary.pdf]

Supplementary data

Table S1. Characteristics of the SARS-CoV-2 mutations from the Reina Fabiola University Clinic (RFUC) genomes

Region (genome)	Mutation	SIFT score[a]	Prediction	ΔΔG (kcal/mol)[b]	S-ACE2 interaction[b]
**ORF1a**	S135R	0,35	Neutral		
	T842I	0,17	Neutral		
	K856R	0,08	Neutral		
	G1307S	0,00	Deleterious		
	L2084I	0,01	Deleterious		
	A2710T	0,01	Deleterious		
	L3027F	0,38	Neutral		
	T3090I	0,52	Neutral		
	L3201F	0,09	Neutral		
	T3255I	0,04	Deleterious		
	P3395H	0,00	Deleterious		
	R3756K	0,37	Neutral		
	I3758V	0,22	Neutral		
**ORF1b**	P314L	0,02	Deleterious		
	R1315C	0,01	Deleterious		
	I1566V	0,07	Neutral		
	T2163I	0,19	Neutral		
**SPIKE**	A27S	0,04	Deleterious		
	A67V	0,51	Neutral		
	T95I	0,00	Deleterious		
	G142D	0,03	Deleterious		
	Y145D	0,01	Deleterious		
	L212I	0,07	Neutral		
	V213G	0,00	Deleterious		
	**R346K**	1,00	Neutral	-0,04	Increase
	**S371F**	0,00	Deleterious	-0,05	Increase
	**S371L**	0,00	Deleterious	0,13	Decrease
	**S375F**	0,27	Neutral	0,18	Decrease
	**T376A**	0,04	Deleterious	0,26	Decrease
	**D405N**	0,03	Deleterious	0,13	Decrease
	**R408S**	0,21	Neutral	0,13	Decrease
	**K417N**	0,00	Deleterious	0,63	Decrease
	**N440K**	0,28	Neutral	0,1	Decrease
	**G446S**	0,04	Deleterious	0,07	Decrease
	**S477N**	0,53	Neutral	-0,23	Increase
	**E484A**	0,02	Deleterious	-0,09	Increase
	**G496S**	0,01	Deleterious	2,17	Decrease

	**Q498R**	0,60	Neutral	1,01	Decrease
	**Y505H**	0,00	Deleterious	0,63	Decrease
	T547K	0,18	Neutral		
	D614G	0,58	Neutral		
	H655Y	0,02	Deleterious		
	N764K	0,00	Deleterious		
	D796Y	0,87	Neutral		
	N856K	0,00	Deleterious		
	Q954H	0,00	Deleterious		
	N969K	0,00	Deleterious		
	L981F	0,54	Neutral		
**ORF3a**	H78Y	0,00	Deleterious		
	L140F	0,00	Deleterious		
	T223I	0,00	Deleterious		
**M**	D3G	0,20	Neutral		
	Q19E	1,00	Neutral		
	A63T	0,24	Neutral		
**ORF6**	D61L	0,00	Deleterious		
**N**	P13L	0,00	Deleterious		
	R203K	0,02	Deleterious		
	G204R	0,00	Deleterious		
	S413R	0,00	Deleterious		

[a] Calculated with SIFT algorithm, [b] Calculated using MutaBind2.

Table S2. Times that each Spike mutation was found in different locations.

**Spike mutations**	**Argentina** (20,974)	**Brazil** (286,735)	**Chile** (46,665)	**Colombia** (24,006)	**Worldwide** (15,000,000)	**Our study** (70)
A27S	23	240	27	8	35506	26
Y145D	1	103	0	3	4,726	25
L212I	0	5	10	2	1618	5

Table S3. HLA alleles used in this study.

Type	Alleles	Frequency	Type	Alleles	Frequency
HLA I	A*01:01	10.28	HLA II	DRB1*01:01	9.87
	A*02:01	21.83		DRB1*03:01	8.93
	A*03:01	9.07		DRB1*07:01	12.6
	A*11:01	8.08		DRB1*11:01	5.94
	A*24:02	10.18		DRB1*11:04	5.23
	A*31:01	6.27		DRB1*13:01	5.94
	B*07:02	5.25		DQB1*02:01	8.9
	B*08:01	6.39		DQB1*02:02	10.94
	B*35:01	6.21		DQB1*03:01	20.31
	B*44:03	6.04		DQB1*03:02	13.72
	B*51:01	7.88		DQB1*06:02	6.73
	C*04:01	15.96		DQB1*06:03	5.98
	C*06:02	7.81		DRB1*15:01	6.62
	C*07:01	14.16			
	C*12:03	6.59			

Table S4. Accession numbers of SARS-CoV-2 genomes are publicly accessible through the GISAID platform (https://www.gisaid.org/).

hCoV-19/Argentina/CBA-CIBICI-01/2021	hCoV-19/Argentina/CBA-CIBICI-01/2022
hCoV-19/Argentina/CBA-CIBICI-02/2021	hCoV-19/Argentina/CBA-CIBICI-02/2022
hCoV-19/Argentina/CBA-CIBICI-03/2021	hCoV-19/Argentina/CBA-CIBICI-03/2022
hCoV-19/Argentina/CBA-CIBICI-48/2021	hCoV-19/Argentina/CBA-CIBICI-04/2022
hCoV-19/Argentina/CBA-CIBICI-04/2021	hCoV-19/Argentina/CBA-CIBICI-05/2022
hCoV-19/Argentina/CBA-CIBICI-05/2021	hCoV-19/Argentina/CBA-CIBICI-06/2022
hCoV-19/Argentina/CBA-CIBICI-06/2021	hCoV-19/Argentina/CBA-CIBICI-07/2022
hCoV-19/Argentina/CBA-CIBICI-07/2021	hCoV-19/Argentina/CBA-CIBICI-08/2022
hCoV-19/Argentina/CBA-CIBICI-08/2021	hCoV-19/Argentina/CBA-CIBICI-09/2022
hCoV-19/Argentina/CBA-CIBICI-09/2021	hCoV-19/Argentina/CBA-CIBICI-10/2022
hCoV-19/Argentina/CBA-CIBICI-10/2021	hCoV-19/Argentina/CBA-CIBICI-11/2022
hCoV-19/Argentina/CBA-CIBICI-11/2021	hCoV-19/Argentina/CBA-CIBICI-12/2022
hCoV-19/Argentina/CBA-CIBICI-12/2021	hCoV-19/Argentina/CBA-CIBICI-13/2022
hCoV-19/Argentina/CBA-CIBICI-13/2021	hCoV-19/Argentina/CBA-CIBICI-14/2022
hCoV-19/Argentina/CBA-CIBICI-14/2021	hCoV-19/Argentina/CBA-CIBICI-15/2022
hCoV-19/Argentina/CBA-CIBICI-15/2021	hCoV-19/Argentina/CBA-CIBICI-16/2022
hCoV-19/Argentina/CBA-CIBICI-16/2021	hCoV-19/Argentina/CBA-CIBICI-17/2022
hCoV-19/Argentina/CBA-CIBICI-47/2021	hCoV-19/Argentina/CBA-CIBICI-18/2022
hCoV-19/Argentina/CBA-CIBICI-17/2021	hCoV-19/Argentina/CBA-CIBICI-19/2022
hCoV-19/Argentina/CBA-CIBICI-18/2021	hCoV-19/Argentina/CBA-CIBICI-20/2022
hCoV-19/Argentina/CBA-CIBICI-19/2021	hCoV-19/Argentina/CBA-CIBICI-21/2022
hCoV-19/Argentina/CBA-CIBICI-20/2021	hCoV-19/Argentina/CBA-CIBICI-22/2022
hCoV-19/Argentina/CBA-CIBICI-21/2021	hCoV-19/Argentina/CBA-CIBICI-23/2022
hCoV-19/Argentina/CBA-CIBICI-22/2021	hCoV-19/Argentina/CBA-CIBICI-24/2022
hCoV-19/Argentina/CBA-CIBICI-23/2021	hCoV-19/Argentina/CBA-CIBICI-25/2022
hCoV-19/Argentina/CBA-CIBICI-24/2021	hCoV-19/Argentina/CBA-CIBICI-26/2022
hCoV-19/Argentina/CBA-CIBICI-25/2021	hCoV-19/Argentina/CBA-CIBICI-27/2022
hCoV-19/Argentina/CBA-CIBICI-26/2021	hCoV-19/Argentina/CBA-CIBICI-28/2022
hCoV-19/Argentina/CBA-CIBICI-27/2021	hCoV-19/Argentina/CBA-CIBICI-29/2022
hCoV-19/Argentina/CBA-CIBICI-28/2021	hCoV-19/Argentina/CBA-CIBICI-30/2022
hCoV-19/Argentina/CBA-CIBICI-29/2021	hCoV-19/Argentina/CBA-CIBICI-31/2022
hCoV-19/Argentina/CBA-CIBICI-30/2021	hCoV-19/Argentina/CBA-CIBICI-32/2022
hCoV-19/Argentina/CBA-CIBICI-31/2021	hCoV-19/Argentina/CBA-CIBICI-33/2022
hCoV-19/Argentina/CBA-CIBICI-32/2021	hCoV-19/Argentina/CBA-CIBICI-34/2022
hCoV-19/Argentina/CBA-CIBICI-33/2021	hCoV-19/Argentina/CBA-CIBICI-35/2022
hCoV-19/Argentina/CBA-CIBICI-34/2021	hCoV-19/Argentina/CBA-CIBICI-36/2022
hCoV-19/Argentina/CBA-CIBICI-35/2021	hCoV-19/Argentina/CBA-CIBICI-37/2022

hCoV-19/Argentina/CBA-CIBICI-36/2021<br>hCoV-19/Argentina/CBA-CIBICI-37/2021<br>hCoV-19/Argentina/CBA-CIBICI-38/2021<br>hCoV-19/Argentina/CBA-CIBICI-39/2021<br>hCoV-19/Argentina/CBA-CIBICI-40/2021<br>hCoV-19/Argentina/CBA-CIBICI-41/2021<br>hCoV-19/Argentina/CBA-CIBICI-42/2021<br>hCoV-19/Argentina/CBA-CIBICI-43/2021<br>hCoV-19/Argentina/CBA-CIBICI-44/2021<br>hCoV-19/Argentina/CBA-CIBICI-45/2021<br>hCoV-19/Argentina/CBA-CIBICI-46/2021	hCoV-19/Argentina/CBA-CIBICI-38/2022<br>hCoV-19/Argentina/CBA-CIBICI-39/2022<br>hCoV-19/Argentina/CBA-CIBICI-40/2022<br>hCoV-19/Argentina/CBA-CIBICI-41/2022<br>hCoV-19/Argentina/CBA-CIBICI-42/2022<br>hCoV-19/Argentina/CBA-CIBICI-43/2022<br>hCoV-19/Argentina/CBA-CIBICI-44/2022<br>hCoV-19/Argentina/CBA-CIBICI-45/2022<br>hCoV-19/Argentina/CBA-CIBICI-46/2022<br>hCoV-19/Argentina/CBA-CIBICI-47/2022<br>hCoV-19/Argentina/CBA-CIBICI-48/2022<br>hCoV-19/Argentina/CBA-CIBICI-49/2022<br>hCoV-19/Argentina/CBA-CIBICI-50/2022

Table S5. Acknowledgment to GISAID contributors (2021-12-03 to 2022-10-22)

Data Availability

GISAID Identifier: EPI_SET_240711rg

doi: 10.55876/gis8.240711rg

All genome sequences and associated metadata in this dataset are published in GISAID's EpiCoV database. To view the contributors of each individual sequence with details such as accession number, Virus name, Collection date, Originating Lab and Submitting Lab and the list of Authors, visit 10.55876/gis8.240711rg

Data Snapshot

- EPI_SET_240711rg is composed of 966 individual genome sequences.
- The collection dates range from 2021-12-03 to 2022-10-22;
- Data were collected in 1 countries and territories;
- All sequences in this dataset are compared relative to hCoV-19/Wuhan/WIV04/2019 (WIV04), the official reference sequence employed by GISAID (EPI_ISL_402124). Learn more at https://gisaid.org/WIV04.

Table S6. Acknowledgment to GISAID contributors (2022-12-14 to 2024-02-17)

Data Availability

GISAID Identifier: EPI_SET_241124wh

doi: 10.55876/gis8.241124wh

All genome sequences and associated metadata in this dataset are published in GISAID's EpiCoV database. To view the contributors of each individual sequence with details such as accession number, Virus name, Collection date, Originating Lab and Submitting Lab and the list of Authors, visit 10.55876/gis8.241124wh

Data Snapshot

- EPI_SET_241124wh is composed of 120 individual genome sequences.
- The collection dates range from 2022-12-14 to 2024-02-17;
- Data were collected in 1 countries and territories;
- All sequences in this dataset are compared relative to hCoV-19/Wuhan/WIV04/2019 (WIV04), the official reference sequence employed by GISAID (EPI_ISL_402124). Learn more at https://gisaid.org/WIV04.

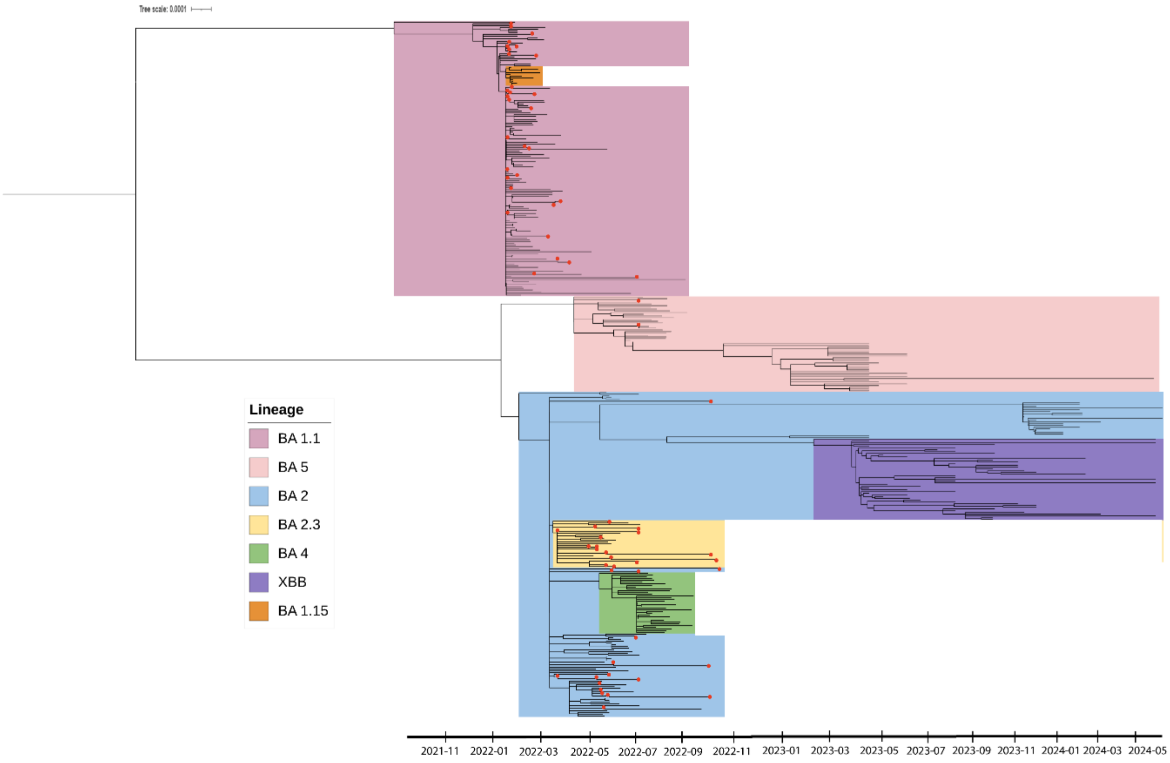

Figure S1. Temporal phylogenetic tree of SARS-CoV-2 isolates updated to 2024. The tree shows the evolutionary relationships and the emergence of the Omicron XBB variant, reflecting ongoing viral evolution. Our samples isolated at the RFUC are indicated with red dots, while the others correspond to the GISAID sequences sampled from the rest of the country. Branch lengths represent genetic distance in terms of substitutions per site, with bootstrap values (500 replicates) indicating branch support. Colors represent different clades of the phylogenetic tree.

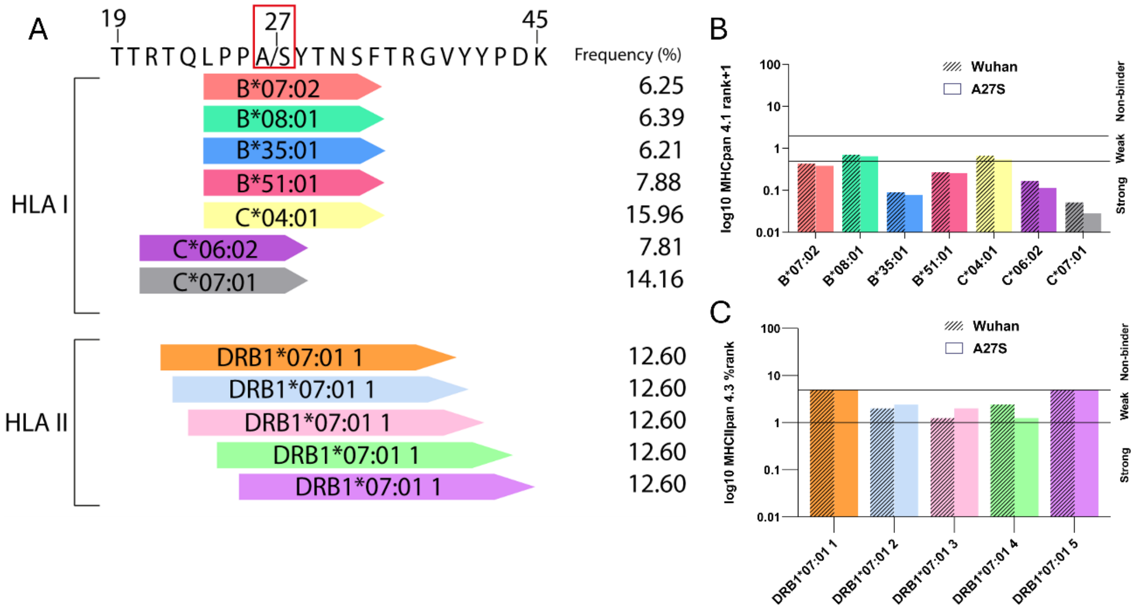

Figure S2. T-cell epitope distribution in the A27S mutation. (A) T-cell epitope distribution between the positions 19 and 45 of the spike protein on its wild-type version. (B) T-cell epitope distribution between positions 19 and 45 of the spike protein on the A27S spike mutant.

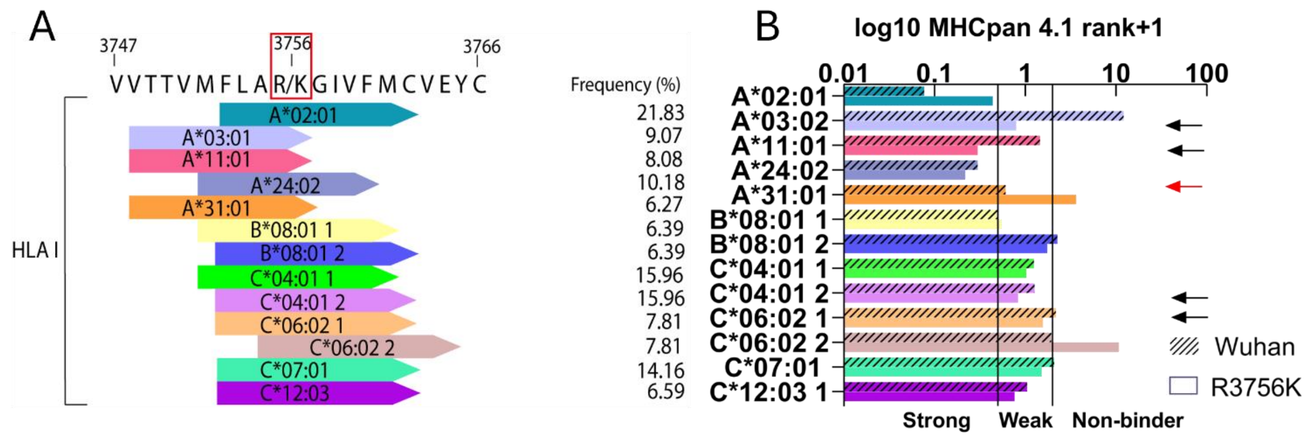

Figure S3. T-cell epitope changes induced by the R3756K mutation in the Orf1a protein. (A) T-cell epitope distribution between positions 3747 and 3766 of the spike protein. (B) T-cell epitope distribution between the positions 3747 and 3766 of the Orf1a protein. (C) Predicted binding affinity of HLA-I molecules between positions 3747 and 3766 of the Orf1a protein. Black arrows indicate alleles where the mutation increases the predicted binding affinity, while red arrows indicate cases where the mutation reduces the predicted binding affinity.
